# A Novel User Control for Lower Extremity Rehabilitation Exoskeletons

**DOI:** 10.3389/frobt.2020.00108

**Published:** 2020-09-08

**Authors:** Kiran K. Karunakaran, Kevin Abbruzzese, Ghaith Androwis, Richard A. Foulds

**Affiliations:** ^1^Kessler Foundation, West Orange, NJ, United States; ^2^Department of Biomedical Engineering, New Jersey Institute of Technology, Newark, NJ, United States; ^3^Stryker Corporation, Mahwah, NJ, United States; ^4^Really Useful Robots, LLC, Langhorne, PA, United States

**Keywords:** lower extremity exoskeletons, gait, spinal cord injury, rehabilitation robotics, robot control systems

## Abstract

Lower extremity exoskeletons offer the potential to restore ambulation to individuals with paraplegia due to spinal cord injury. However, they often rely on preprogrammed gait, initiated by switches, sensors, and/or EEG triggers. Users can exercise only limited independent control over the trajectory of the feet, the speed of walking, and the placement of feet to avoid obstacles. In this paper, we introduce and evaluate a novel approach that naturally decodes a neuromuscular surrogate for a user's neutrally planned foot control, uses the exoskeleton's motors to move the user's legs in real-time, and provides sensory feedback to the user allowing real-time sensation and path correction resulting in gait similar to biological ambulation. Users express their desired gait by applying Cartesian forces via their hands to rigid trekking poles that are connected to the exoskeleton feet through multi-axis force sensors. Using admittance control, the forces applied by the hands are converted into desired foot positions, every 10 milliseconds (ms), to which the exoskeleton is moved by its motors. As the trekking poles reflect the resulting foot movement, users receive sensory feedback of foot kinematics and ground contact that allows on-the-fly force corrections to maintain the desired foot behavior. We present preliminary results showing that our novel control can allow users to produce biologically similar exoskeleton gait.

## Introduction

Individuals with complete paraplegia due to spinal cord injury (SCI) have impaired motor control and sensory feedback that limits their ability to walk (Shepherd Center). While wheelchairs provide alternative mobility to individuals with paraplegia, they are not a complete substitute for natural ambulation. Current research has addressed this issue with wearable lower extremity exoskeletons (Dollar and Herr, 2008; Contreras-Vidal et al., 2016). The past decade has witnessed a dramatic growth in the study and implementation of such technology, not only for those with paraplegia due to SCI, but also for individuals with cerebral palsy, stroke, traumatic brain injury and multiple sclerosis (Canela et al., 2013; Murray et al., 2015; Kozlowski et al., 2017; Lerner et al., 2017; Patané et al., 2017; Androwis et al., 2019; Karunakaran et al., 2019). These exoskeletons are mechanically similar, consisting of a set of linkages that parallel the wearer's thighs, calves, and feet, and augmented with actuators to provide alternatives to muscle torque at the joints. Unlike devices developed for military and industrial tasks, most rehabilitation exoskeletons rely on the subject's use of crutches or canes to provide balance, as the devices lack sensory feedback and balance compensation (Contreras-Vidal et al., 2016).

Where current rehabilitation exoskeletons differ significantly is in their detection of user initiation of gait patterns (Dellon and Matsuoka, 2007; Strickland, 2012)^1^^,^^2^. The Ekso (Ekso Bionics) has motors at the hip and knee, with passive springs at the ankles to provide gait only in the sagittal plane (Strickland, 2012)^1^. The Ekso has two options to initiate the gait cycle. (1) The first allows a clinician to control gait by means of an external switch pad for training and therapy. (2) For independent control, sensors embedded in the device detect changes in the hip position^1^. A step can be initiated by the user moving the hip forward and laterally or by changing tilt angle and making ground contact with sensors on the crutches^1^. Goldfarb et al. developed what has been commercialized as the Indego (Parker Hannifin) (Farris, 2012; Farris et al., 2012; Quintero et al., 2012). It also has powered degrees of freedom (DOF) at the hip and knee, and passive ankle support in the sagittal plane. It uses Hall effect sensors, potentiometers, and accelerometers to detect the user's center of pressure (COP) (Farris, 2012; Quintero et al., 2012). When the user leans forward with both crutches touching the ground, the COP shifts in the direction of movement, and the exoskeleton initiates swing of the most rearward leg (Farris, 2012; Quintero et al., 2012). The Rewalk (Rewalk Robotics) also has two active DOFs with the ankle consisting of a simple orthotic joint with limited motion and spring assisted dorsiflexion (Esquenazi et al., 2012). The control system includes a tilt sensor to determine changes in trunk motion and center of gravity. Shifts in the center of gravity initiate the preprogrammed hip and knee displacement in the appropriate leg (Esquenazi et al., 2012). The HAL (Cyberdyne) employs a combination of EMG gait initiation detection with an accelerometer and gyroscope to sense body posture (Lee and Sankai, 2002, 2003; Hayashi et al., 2005). In contrast to the above exoskeletons that have two active degrees of freedom, the REX (REX Bionics) has 5 motors per leg and is the only available assistive exoskeleton to provide movement in the coronal as well as sagittal planes, and to be self-balancing (i.e., no crutches^2^). It is controlled by a joystick that signals one of 8 discrete directions of ambulation, and has button selection for sitting and rising^2^.

Electrophysiological signals have also been employed to initiate exoskeleton gait (Kilicarslan et al., 2013; Kwak et al., 2015; Lebedev and Nicolelis, 2017). Contreras-Vidal et al. have demonstrated the use of EEG triggers to select various REX exoskeleton's discrete control commands (Farris et al., 2012). Similarly, other investigators have focused on detecting gait initiation and termination events using EEG, EOG, evoked potentials, and other bioelectric signals (Nicolelis, 2003; Kilicarslan et al., 2013; Kwak et al., 2015).

Beyond the triggering of preplanned gait patterns, extensive research has been pursued on control methods that allow users to plan and execute novel gait patterns similar to those of individuals with no disability. Gancet et al. have tried to interpret EEG signals from the motor cortex to calculate the kinematics of the gait cycle. A dynamic recurrent neural network was used to train the network to detect the gait patterns in the EEG signal (Gancet et al., 2012). Lebedev and Nicolelis (2017) has also attempted to use BCI to communicate the user's desired gait cycle kinematics. Unfortunately, both groups have reported numerous challenges such as difficulty in identifying the user intention of each joint, removal of mechanical artifacts caused by relative movement of the EEG cap, and physiological artifacts due to muscle activity in the vicinity of the cap. Even with extensive signal processing, they were not able to completely isolate the relevant signals at all time periods. López-Larraz et al. (2016) have concluded that the current state of the art of non-invasive BCI knowledge is insufficient for precise decoding of neutrally intended leg kinematics.

Similar to our intention of redirecting controllable neuromuscular activities to define real-time novel gait patterns, Durandau et al. (2019) have explored the use of residual muscle force activity by using detecting EMG signals. These investigators explain that no other exoskeleton has the ability to amplify weak user muscle strength. Another group (Ferris and Lewis, 2009) have considered the use of proportional EMG signals to activate pneumatic muscles that power a lower extremity exoskeleton. Yet another group has recognized the contralateral synchronization of the arms and legs in unimpaired walking and has used the shoulder angles to define unique sets of hip, knee, and ankle angles that are provide to the exoskeleton (Fang et al., 2017).

Similar to those efforts, the long-term objective of our work is to allow users to intuitively express their desired gait kinematics and dynamics using their arms and hands as sensory and motor alternatives to their legs and feet. Our prior lower extremity research (Karunakaran et al., 2014, 2017) evaluated the feedback conditions required by the hand to produce gait kinematics. That study included 18 subjects controlling virtual feet using hand movements to produce gait trajectories in a virtual environment. Our results indicated that users, provided with haptic through a physical link, and visual feedback (both sensations felt by the hands and visual observation), produced hand and virtual foot trajectories similar to biological gait trajectories (Karunakaran et al., 2014, 2017). We showed that for this to be a viable exoskeleton control method, the hands must be haptically connected either contralaterally or ipsilaterally to the feet. This ensures that the hands and feet move in precise time synchrony, and that hands sense the movement of the feet in order to provide the central nervous system with both position and force feedback from the feet (Karunakaran et al., 2014, 2017). This study as well as our other prior work has shown that neural control of arm and finger walking-like movements generates kinematics very similar to biologically intact neurally determined leg movements and foot placement (DeMarco and Foulds, 2002; Birmingham et al., 2003; Karunakaran et al., 2014, 2017).

The goal of this work was to develop and user test an intuitive control mechanism that independently controls both legs, while producing symmetrical gait kinematics using trajectories generated by hand movements in real time. Balance and co-ordination are being addressed in the later phase of the work, and will be presented in a subsequent paper.

We have chosen to allow the user to control movement of each foot with movements of the ipsilateral hand and arm. This may appear counter intuitive since the arms swing contralaterally with respect to leg movements during normal walking. However, we believe that ipsilateral control presents little if any physiological impediment to a successful user/exoskeleton interface, while its advantages are significant.

Although contralateral arm swing is commonly observed, its function in human gait is not entirely understood. Recent studies (Meesen et al., 2006; Meyns et al., 2013) agree there may be an enhancement in balance and stability, however, they also note that walkers can carry objects and make purposeful arm movements without compromising balance.

Contralateral arm movement is the preferred pattern at walking speeds above 0.8 m/s, yet at speeds below 0.8 m/s, individuals will often adopt an ipsilateral pattern or not swing the arms at all (Ford et al., 2007). In studies of walking with constrained or impaired arm movements (Meesen et al., 2006; Ford et al., 2007) there were only minor reductions in walking speed, that could be voluntarily corrected by participants. Studies of ladder climbing (Armstrong et al., 2009) show a preference for ipsilateral hand and arm coordination. Most importantly, in Meesen et al. (2006) subjects were asked to make four types of simultaneous arm/leg movements: ipsilateral same direction movements (e.g., right arm and right leg raised up and down, or adducted/abducted), opposite direction ipsilateral movements (e.g., right arm up while right leg down, or right arm adducted and right leg abducted), contralateral same direction movements and contralateral opposite direction movements. When examining the quality to interlimb coordination, the investigators found that the ipsilateral, same direction movements were modestly more accurate in absolute position/angle and phase than both contralateral conditions (Meesen et al., 2006). Thus, we are confident that we could employ either ipsilateral or contralateral arm/hand control method.

For our purposes, ipsilateral control of foot movement has several significant advantages. Admittance control requires the user to apply forces to the foot of the exoskeleton. This is most easily accomplished via a rigid trekking pole on the same side as the foot. A pole on the same side of the body facilitates directing the foot to move vertically and horizontally in the sagittal plane as well as controlling ab/adduction in coronal plane. Also, the poles allow the user's to feel the impact of the ground reaction through the ipsilateral hands. This quality of sensory feedback is unavailable in any proposed exoskeleton control method, and has been shown in our earlier work (Karunakaran et al., 2017) to be of vital importance to controlling the movement of the feet.

## Materials and Methods

### Apparatus

A 1/2 scale biped robot representing a lower extremity exoskeleton was built to test our control method. Each leg has 2 links, from hip to knee, and from knee to ankle, and a foot scaled to the anthropometry of the human leg.

**Figure 1:**
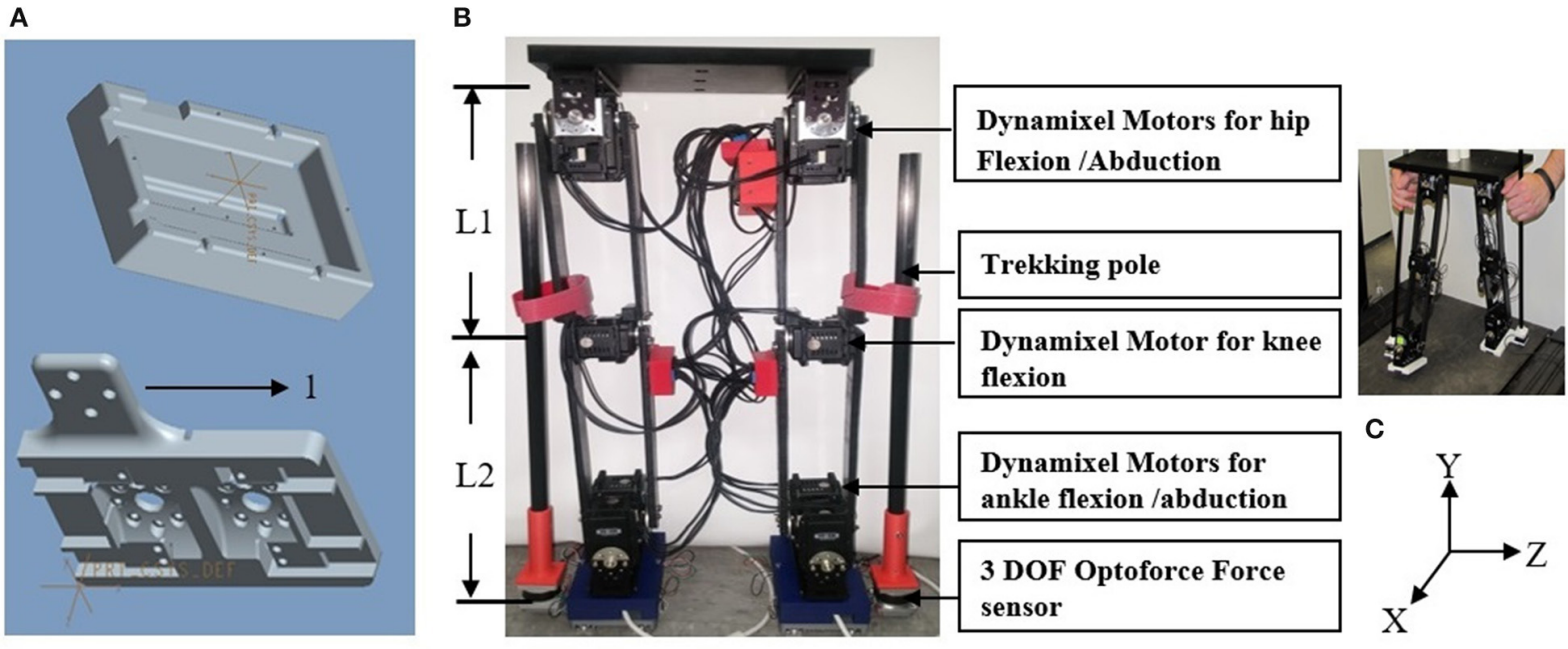
**(A)** Mount to attach the foot to ankle motors. **(B)** Foot of the biped with extrusion to mount (1) Optoforce. **(C)** Front view of 10 DOF biped robot designed based on anthropometric data. L1 is the link length between hip and knee, L2 is link length between knee and foot. **(C)** The coordinate system X, Y, and Z used for robot's movement on treadmill.

Each robot leg has 5 DOF (Figure 1). The hip has 2 DOF for flexion/extension and abduction/adduction, the knee has 1 DOF for flexion/extension, and the ankle has 2 DOF for plantarflexion/dorsiflexion and inversion/eversion. Using previously published data (Hamill et al., 2013), maximum thigh angular velocities and angular accelerations were found to be 28 radians/second (rad/s) and 35 radians/second^2^, respectively. Using this angular acceleration with an estimate of the robot leg moment of inertia relative to the hip, the motor torque required to achieve the maximum acceleration was computed to be 4.2 Newton-meter (N-m). The similarly computed maximum knee motor torque is smaller due to the smaller moment of inertia. We selected the Dynamixel MX-106 smart servomotor (Robotis, USA)^3^ as the actuator for all joints, since both its angular velocity, 42 rad/s, resolution of 0.088 degrees and maximum torque, 8.5 N-m, allow the robot to match the physiological leg segment velocities and accelerations.

Dynamixel servos employ Maxon motors supported by 32-bit internal microcontrollers providing proportional/integrative/derivative (PID) control at 1,000 Hz. All motors are daisy chained by a 3-wire bus on which they are group addressed from MATLAB software at 1 Mbits/second so that the motors are activated simultaneously. A 3 DOF Optoforce sensor^4^ detects the forces exerted by the user on carbon-fiber trekking poles that are rigidly attached to the sensor.

### User Control Algorithm

The control algorithm consists of an outer admittance loop running at 100 Hz and an inner impedance loop running at 1,000 Hz. The Dynamixel motor's internal PID controller serves as the inner loop. Our admittance loop receives the Cartesian forces applied to the trekking poles by the user's hands and generates desired Cartesian kinematics for the end-effectors of the robot every 10 milliseconds (ms). Admittance control offers a very intuitive control mechanism; where the robot end-effector will be directed to move in the Cartesian directions proportional to the force applied by the user (Van Der Linde et al., 2002). The force can be scaled to accommodate the user's needs. It is safe and easy for human interaction (Van Der Linde et al., 2002; Haidegger et al., 2009).

Custom inverse kinematics algorithms transform the Cartesian positions generated by the admittance loop into joint angles used to command the motors. Algorithms are written in MATLAB, with time-dependent functions coded in C to maintain a 100 Hz update rate.

### Sagittal Plane Control

**Figure 2:**
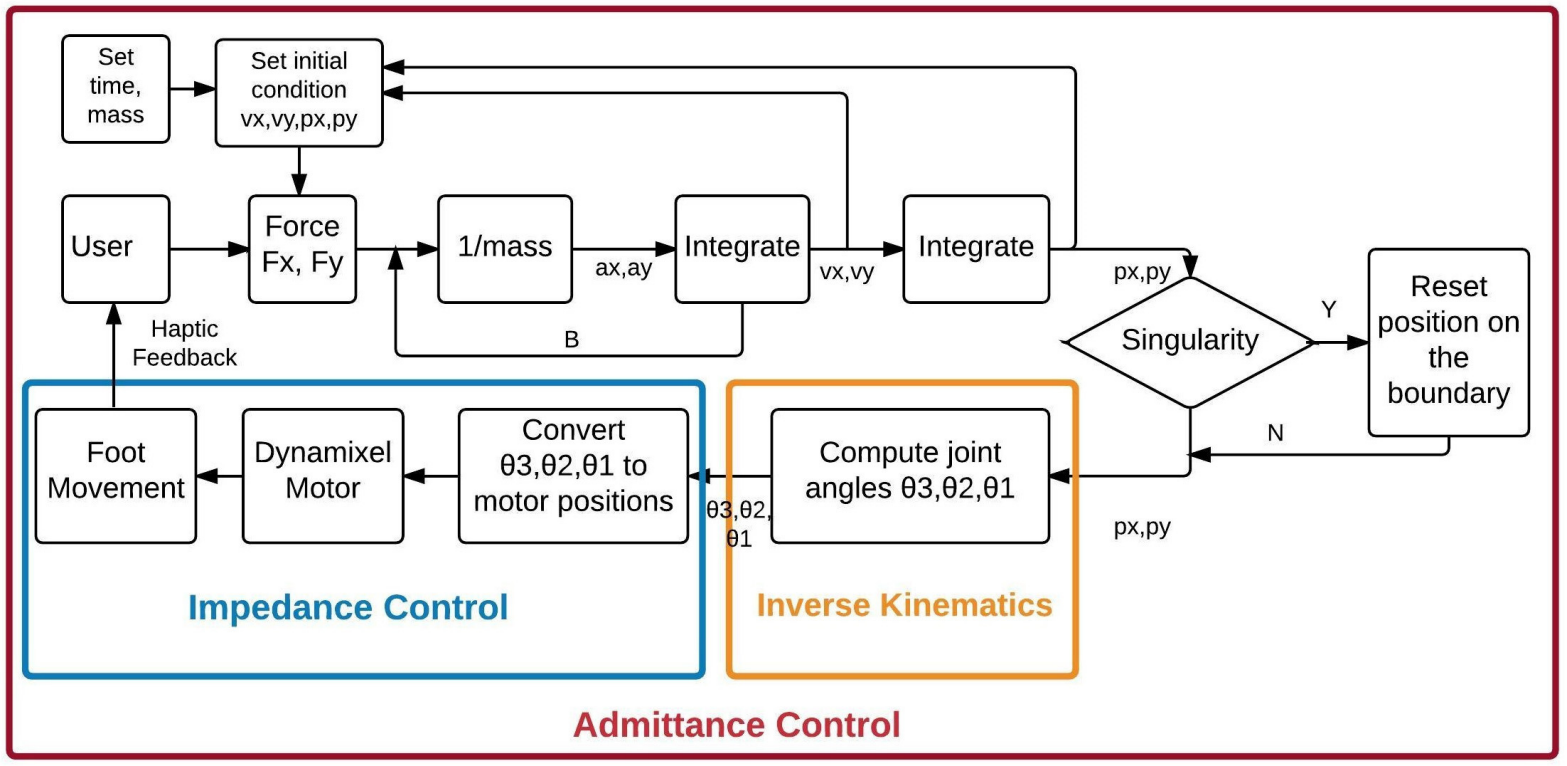
The Admittance Control algorithm for control of biped gait in the sagittal plane. θ1 is hip angle, θ2 is knee angle, and θ3 is the ankle angle computed based on Equations (1-3), respectively. Velocities in X and Y are represented by vx and vy, respectively. Acceleration in X and Y are represented by ax, and ay, respectively. Position in X and Y are represented by px and py, respectively.

Robot control in the sagittal plane is shown in Figure 2. User forces applied in the X and Y-direction are read at 1,000 Hz from two axes of the 3 DOF Optoforce force sensor, and averaged to provide 100 samples/second. For every cycle of the admittance loop these forces, virtual mass, virtual damping, as well as the foot's Cartesian position and velocity are passed to custom-written ordinary differential equation (ODE) that is implemented using the C source code Variable ordinary differential equation (CVode) solver and compiled for use in MATLAB (Van Riel, 2012). The ODE is shown in Figure 2 as a double integration that provides the desired positions and velocities in X and Y to be achieved in the next 10 ms. The value assigned to the mass allows scaling of the forces to meet the capabilities of the user's hands. The ODE also incorporates the admittance loop's damping that maintains stability of the system. No admittance stiffness is included in this version of the software, but can be added if required in future situations. The ODE function is coded to solve the second-order differential equation shown below:

(1) $$\begin{array}{l} {\text{X}}^{\prime \prime }\left(\text{t}\right)=\frac{\text{F}\left(\text{X}\right)}{\text{M}}-\frac{\text{B}*{\text{X}}^{\prime }\left(\text{t}\right)}{\text{M}} \end{array}$$

where, F(X) = force (Newton), M = virtual mass (kilogram), B = virtual damping (Newton-second/meter), X′ (t) =velocity (meter/second), X″ (t) = acceleration (meter/second^2^).

### Inverse Kinematics

Since the robot has revolute joints, inverse kinematics converts the X-Y position of the foot to angles of the hip and knee in the sagittal plane. For simplicity, the angle of the ankle is computed to keep the foot parallel to the floor. The angles are calculated using the law of cosines (Equations 2–4). The joint angles are in turn converted to hip, knee and ankle motor units in the sagittal plane. These values are in turn fed to the corresponding motors to generate the required torque to perform the movement intended by the user.

(2) $$\begin{array}{l} \theta 2=-2tan^{-1}\;\sqrt{\frac{{\left(L1+L2\right)}^{2}-{\left(X1+Y1\right)}^{2}}{{\left(X1+Y2\right)}^{2}-{\left(L1+L2\right)}^{2}}} \end{array}$$

(3) $$\begin{array}{l} \theta 1=\text{tan}\left(\frac{L2sin\theta 2}{L1+L2cos\theta 2}\right)-\text{tan}\left(\frac{Y1}{X1}\right) \end{array}$$

(4) $$\begin{array}{l} \theta 3=\theta 2-\text{tan}\left(\frac{Y1}{X1}\right)+\text{tan}\left(\frac{L2sin\theta 2}{L1+L2cos\theta 2}\right) \end{array}$$

where, X1, Y1 is the desired end-effector position, L1 is the link length between hip and knee, L2 is link length between knee and ankle, θ1 is hip angle, θ2 is knee angle, and θ3 is the ankle angle.

The Dynamixel motors have sufficiently fast mechanical and electrical response times (~4 ms) to relocate the robot end-effector to the desired location within the 10 ms loop period^3^. The new position is read at the beginning of the next admittance cycle and serves as the initial conditions for the ODE. As the user's hand is rigidly connected to the robot foot, the user receives real-time sensation of foot movement. The user can modulate his/her forces to alter the speed of foot movement independently in X and Y, and also in response to external forces that may impede the foot.

### Singularity Check

The algorithm verifies that the predicted X-Y position is within reach of the robot. If this check fails, the next position is set to a location on the boundary of the robot's range on a line between the former and predicted position. This maintains stability and smoothness at the singularity. The robot reaches singularity when the estimated position is outside the range of motion of the robot.

### Coronal Plane Control

**Figure 3:**
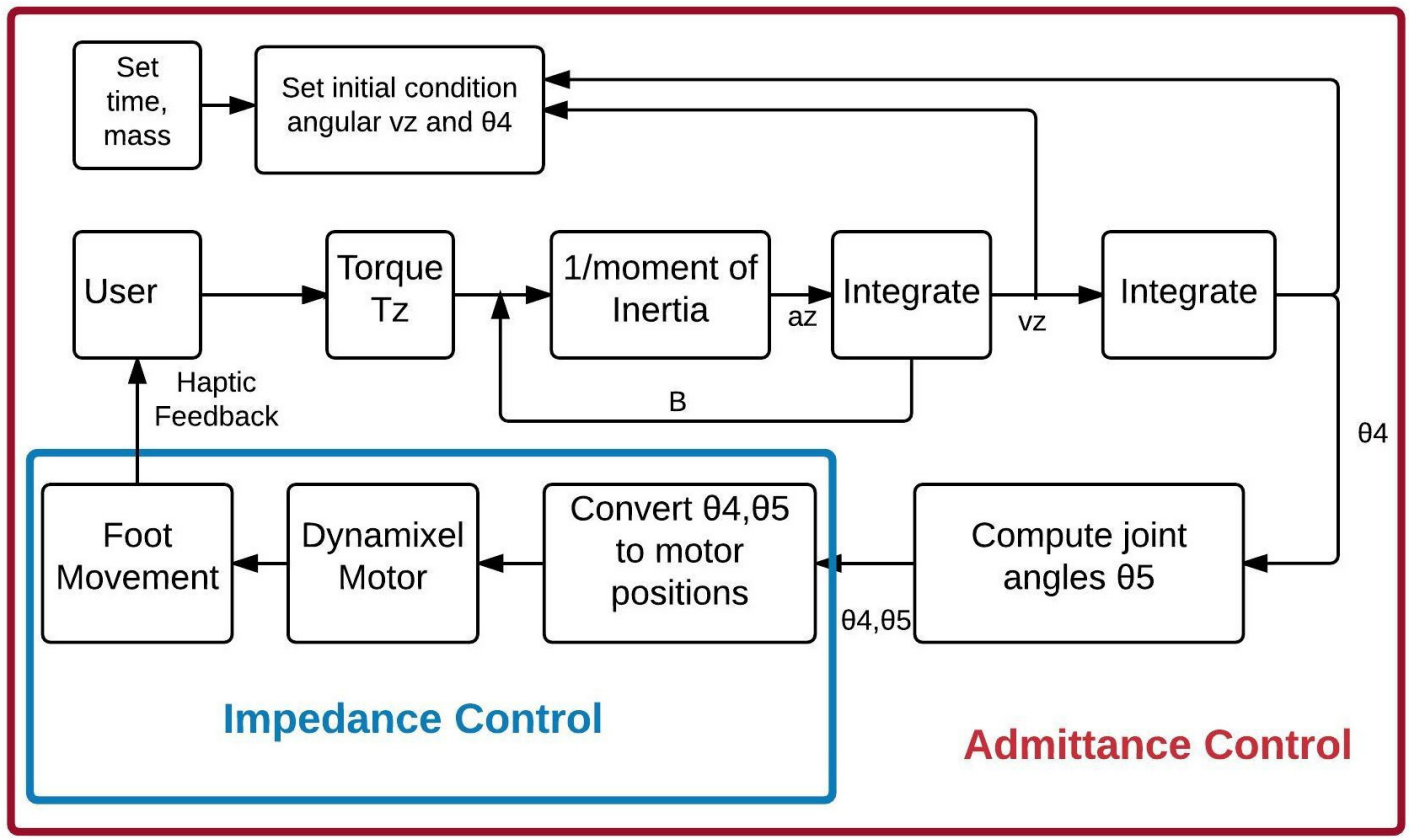
The Admittance Control algorithm for trekking pole control of coronal plane movement of the robot leg. θ4 is hip angle, and θ5 is the ankle angle and is computed using Equations (6) and (7). Angular velocity and angular acceleration is denoted by vz and az, respectively.

Force applied by the user to a trekking pole in the z-direction is similarly converted to rotation of the hip in the coronal plane as shown in Figure 3. The user input is treated as an applied torque, so the ODE implements the following rotational equation of motion:

(5) $$\begin{array}{l} \theta^{\prime \prime }\left(\text{t}\right)=\frac{\text{T}\left(\theta \right)}{I}-\frac{\text{B*}\theta^{\prime }\left(\text{t}\right)}{\text{I}} \end{array}$$

(6) $$\begin{array}{l} \theta 4=\int \theta^{\prime }\left(\text{t}\right) \end{array}$$

(7) $$\begin{array}{l} \theta 5=180-\theta 4 \end{array}$$

where, T(θ) = user applied torque (Newton-meter), I = virtual moment of inertia (kilogram-meter^2^), B = rotational damping (Newton-second/meter), θ′ (t) = angular velocity (meter/second), θ″ (t) = angular acceleration (meter/second^2^). θ4 is hip angle, and θ5 is the ankle angle in the coronal plane.

The ab/adduction angle is executed by the second hip motor, with the new angle serving as the initial condition for the next admittance cycle, and the movement of the robot leg provides a haptic sensation to the user's hand. Similar to the sagittal plane, the inversion/eversion angle of the foot is computed to keep the foot parallel to the floor.

### Biped Control Strategy for Users

**Figure 4:**
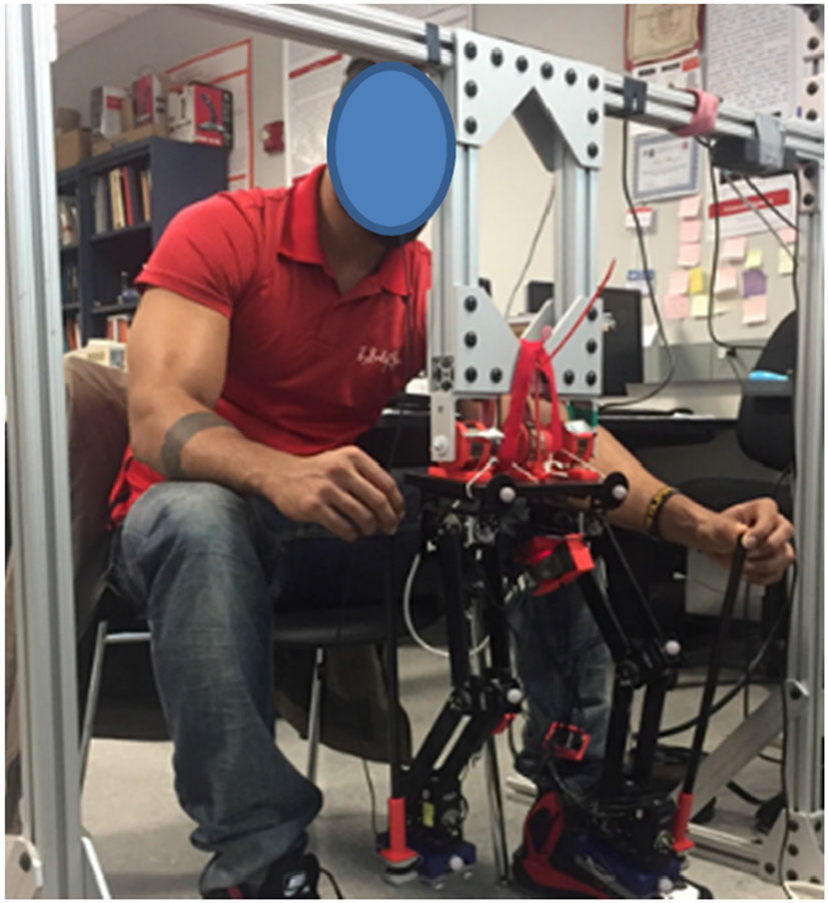
User controlling the biped by holding the trekking poles on the ipsilateral side using hands. Constant force springs connect the robot to the overhead frame to maintain balance.

Our biped control strategy allows the user to execute the swing and the stance phases of each robot leg independently by applying ipsilateral hand forces at the top of the trekking poles, as shown in Figure 4. The user forces are converted to desired foot positions by the admittance control software, with servomotor response occurring within 10 ms.

Forward progression in gait requires activation of both legs. The swing leg proceeds from ground contact (toe-off) to swing and ultimately to a subsequent heel contact in a new forward position on the ground. At the same time, the stance foot remains in the same position on the ground, with rotation of ankle and hip of the stance leg allowing forward progression of the torso (i.e., shifting the robot's center of gravity forward).

The swing foot movement is controlled by the user applying a time-varying upward hand force to define the Y-axis trajectory and a forward force to signify the desired X-axis foot trajectory. Simultaneously, the stance side hand applies a rearward force only in the negative X direction. As this foot remains in contact with the ground (while the swing foot is in the air), the admittance software controls the ankle, knee, and hip motors of the stance leg for forward progression.

Haptic feedback is provided through the physical link (trekking pole) between the hands and the feet. During the swing, as the user force is converted to foot movement, the trekking pole follows the foot, allowing the hand to move synchronously with the trekking poles to walk the biped. Near the end of swing, the user applies downward force to the trekking pole to bring the foot to heel strike. At heel strike, the ground precludes further downward movement, and the user's hand feels the ground reaction.

### Evaluation of the Accuracy of the Control

A slow gait-like movement was performed by one healthy female participant (who was a member of the research team) for a period of 60 s in the air to evaluate the accuracy of the admittance control and inverse kinematics algorithms, and the time delay. This work was approved by the New Jersey Institute of Technology's (NJIT) institutional review board (IRB).

The accuracy of the control algorithm for the sagittal and coronal plane was assessed by comparing the desired and the actual Cartesian position of the feet in X, Y, and Z direction. The “desired position” is the position of the foot computed by the algorithm based on user input, and the “actual position” is the position reached by the foot of the biped. The accuracy of the Dymanixel's internal impedance control was evaluated by comparing the desired and actual joint angle. The accuracy of the hip and knee angles in the sagittal plane was evaluated while the participant performed the gait using the hand. The accuracy of the joint angle of the hip in the coronal plane was evaluated while the participant performed adduction/abduction. The “desired joint angles” are the angles computed by the algorithm, and the “actual angles” are the angles reached by each joint motor. The motor angles at the end of each iteration from each motor were converted to joint angles of the hip and knee. These joint angles were compared to the desired joint angles at every given time point. The accuracy in sagittal plane was evaluated using a forward kinematics algorithm developed to obtain the X and Y positions of the foot. This position was in turn compared with the desired X and Y positions (X and Y position computed using the admittance control) to evaluate the accuracy of the inverse kinematics algorithm.

A Pearson's r correlation was performed to quantify the similarity between the actual and desired joint angles (hip flexion/extension, knee flexion/extension, and hip adduction/abduction) and Cartesian positions (X, Y, and Z positions).

The average time delay was computed as the iteration time (time required to move all the joints by the motor from the time the forces were applied by the user).

### Evaluation of Gait Using the Control Mechanism

After validating the accuracy of the experimental apparatus, data were collected for an extended study evaluating the control mechanism using seven naïve participants who controlled the gait of the biped on a treadmill using control mechanism.

#### Participants

The study included seven naïve participants (2 male and 5 female) for biped walking and one reference participant (control) from whom we collected data on human gait. All participants were between ages 18 and 35, with fully functional upper and lower extremities. Exclusion criteria included any disability to the upper or lower extremities or inability to perform normal gait. The study was approved by the NJIT IRB and the experiment was performed with the participants' consent.

#### Experimental Setup

The experimental setup included a Pro-form J6 treadmill around which a custom frame was built using 80–20 aluminum to support the biped. The frame allowed the users to have complete view of the treadmill and the robot. An Optitrack Trio motion capture system was used to record the biped gait as well as the reference participant gait. As the treadmill was designed for human use, its lowest speed would not accommodate the small-sized robot, thus its speed was reduced when used for biped walking by adding a power resistor in series with the motor. Optitrack markers were placed on the hip, knee, and ankle of both the legs to track the biped gait and the reference participant gait.

#### Biped Walking

The biped was placed on the treadmill and each participant was seated in a comfortable chair behind the treadmill. The participants were instructed to control the gait of the biped during each trial by applying small forces to the pole extending from the sensor on each leg in the direction of the intended movement. The study consisted of eight trials. Each trial lasted 1 min, followed by a 30 s rest. The speed of the treadmill during each trial was varied as shown in Table 1. The speed variations for the biped were 0.1 (low), 0.2 (medium), and 0.3 (high) mph.

**Table 1:** Speed of treadmill for each trial.

**Trial**	**1**	**2**	**3**	**4**	**5**	**6**	**7**	**8**
Speed	Medium	Medium	High	Low	Medium	High	Low	Medium

The participants performed familiarization sessions before the start of the actual session and those data were not included in the analysis. The first familiarization session was performed without the treadmill for 1 min, where the participants controlled the leg of robot in the air to get accustomed to kinematics of the leg. The second familiarization session included eight trials, where each trail lasted 1 min and was performed on the treadmill with the lowest speed. The third familiarization session included controlling the biped at different speeds for a minute each, as shown in Table 1.

The participant from whom the reference gait data were collected for one trial used the same treadmill at a self-selected speed for comfortable walking.

#### Data Analysis

Horizontal and vertical trajectories collected at 120 Hz of the ankle, hip and knee were filtered using a 4th order, zero-lag Butterworth low-pass filter. The filtered data were used for further analysis. The data were further divided into gait cycles.

##### Spatial and temporal symmetry

The foot trajectories in the sagittal plane by 7 participants and 1 reference participant were evaluated for the effectiveness of the control using hand trajectories using temporal and spatial symmetry outcomes.

The swing and stance time of each foot during each gait cycle was computed, and equation 8 was used to compute the temporal symmetry (Patterson et al., 2008). Similarly step length and step height of each foot were computed for each gait cycle, and equation 9 and 10 were used to compute spatial and step height symmetry (Patterson et al., 2008). The average temporal, spatial, and step height symmetries were computed for all participants in each trial.

Statistical analysis was performed on all trials across the seven participants. Shapiro-Wilk test (*p* > 0.05) of normality showed that data were normal for spatial and temporal symmetry. Repeated measures analysis of variance (ANOVA) was performed on the spatial and temporal symmetry, respectively to determine the effect of change in speed on the spatial and temporal symmetry, respectively. Further, a Greenhouse- Geisser test was performed, since the data showed significance with Mauchly's test for sphericity. Shapiro-Wilk test (*p* < 0.05) of normality showed that data were not normal for step height symmetry. Hence, Friedman Test was used to determine the difference between different trials.

(8a) $$\begin{array}{l} Temporal\;swing\;stancesymmetry=(swing\;time)/(stance\;time)\\ \;Overall\;temporal\;symmetry \end{array}$$

(8b) $$\begin{array}{l} =\left(\frac{Right\;temporal\;swing\;stance\;symmetry}{Left\;temporal\;swing\;stance\;symmetry}\right) \end{array}$$

(9) $$\begin{array}{l} Spatial\;symmetry=\left(\frac{Right\;step\;length}{Left\;step\;length}\right) \end{array}$$

(10) $$\begin{array}{l} Step\;Height\;symmetry=\left(\frac{Right\;step\;height}{Left\;step\;height}\right) \end{array}$$

##### Duty cycle

The percentage of stance and swing phase for each gait cycle was calculated using equations 11, 12, and 13. The average duty cycle of all gait cycles was computed for all participants in each trial and for the reference participant.

Statistical Analysis was performed on all trials across the seven participants. Shapiro-Wilk test (*p* > 0.05) of normality showed that data were normal. Mixed design analysis of variance (ANOVA) was performed on the stance and swing duty cycle, respectively to determine the effect of change in speed as well difference between left and right leg on the stance and swing duty cycle, respectively. The Greenhouse-Geisser test was used, since the data showed significance with Mauchly's test for sphericity to determine the effect of change in speed.

(11) $$\begin{array}{l} Duty\;cycle=Stance\;Phase+Swing\;Phase \end{array}$$

(12) $$\begin{array}{l} Stance\;Phase\%=100\;*\;Stance\;phase/Duty\;Cycle \end{array}$$

(13) $$\begin{array}{l} Swing\;Phase\%=100\;*\;Swing\;phase/Duty\;Cycle \end{array}$$

##### Joint angles

The joint angles of hip and knee of both the legs of the biped robot walking for all participants were computed from the filtered Cartesian position from the Optitrack data using inverse kinematics. The joint angles were also computed for the gait of the single reference participant.

We statistically compared the similarity of the hip and knee angles of all seven participants with those of the reference participant by computing their Pearson's r correlation after the data had been time-warped to allow a direct comparison (Kianimajd et al., 2017). We followed the method described in La Scaleia et al. (2014).

##### User feedback

Subjective user feedback was obtained from all 7 participants after completing the study. The following questions elicited user experience of the control technique and the responses to each were averaged across all participants.

1. Was it easy, moderate or difficult to use hand movements as control? 0 being easy and 10 being difficult.
2. How tired were your hands after each session? 0 being not tired at all and 10 being very tired.
3. Was using hands to the control the leg intuitive? 0 being least intuitive and 10 being very intuitive.
4. How much force was required to move the leg in the direction intended? 0 being least force and 10 being great force.
5. Did you feel the haptic feedback every time foot made contact with the floor? Yes or No.

## Results

### Accuracy of the Foot Positions and Joint Angles

**Figure 5:**
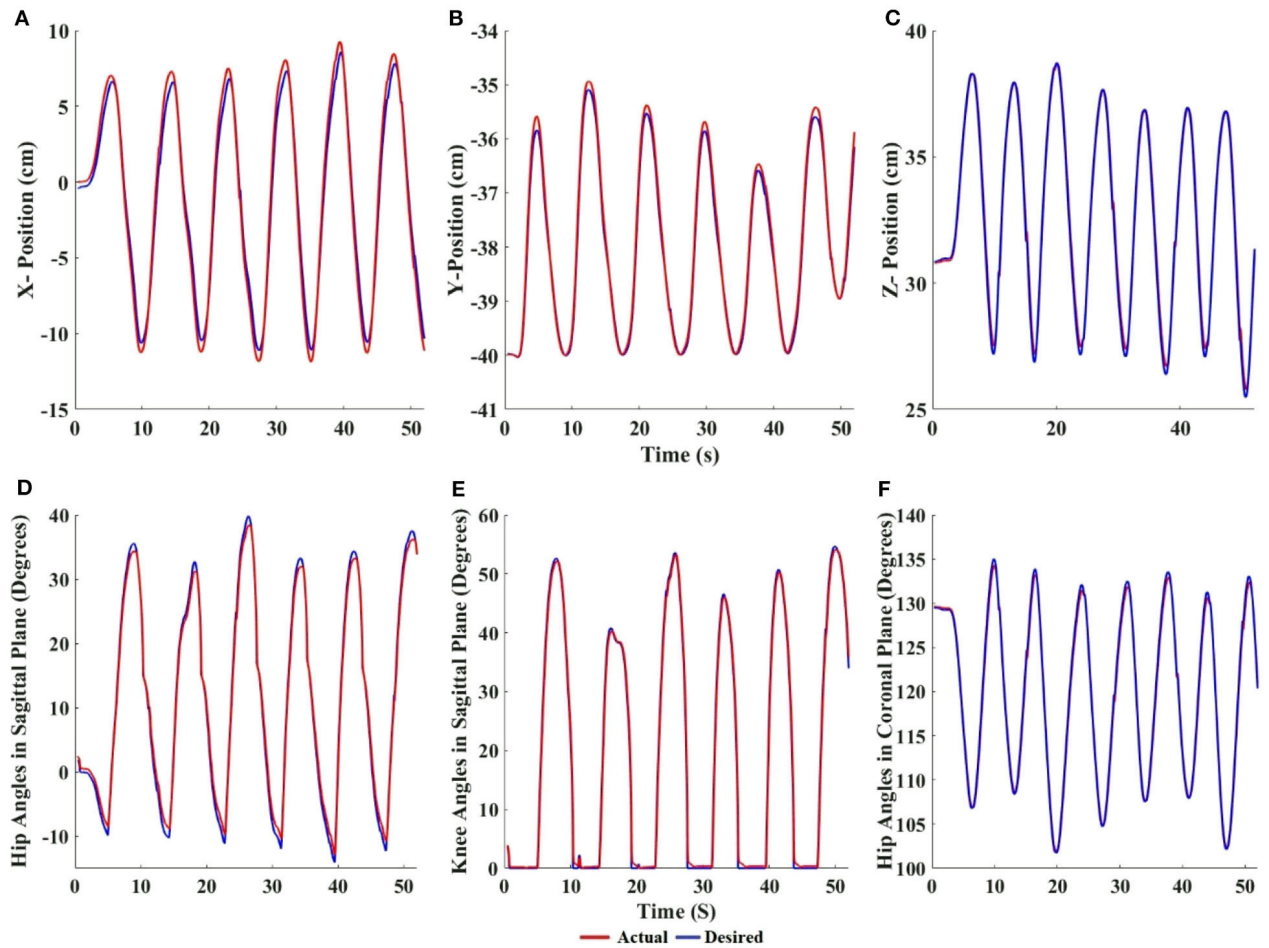
Cartesian values of the desired and actual foot position of **(A)** X, **(B)** Y, and **(C)** Z positions vs. time for the left foot. Desired angles generated by the inverse kinematics and actual angles achieved by the motors of **(D)** hip in sagittal, **(E)** knee in sagittal, and **(F)** hip in coronal plane vs. time for left leg. The flat regions in the knee plot indicate the stance phase of gait. The red lines denote the actual position/angle reached by the robot and the blue lines denote the desired position/angle computed by the algorithm.

Figures 5A–C show the actual and desired Cartesian positions of X, Y (sagittal plane), and Z (coronal plane), respectively, for a single evaluator of the control mechanism of the robot. The results exhibit minimal positional lag. This is quantitatively shown in Table 2, where the mean positional lag (difference between actual and desired position) is <1 cm in the X, Y, and Z-directions, with Pearson's r showing high correlation between the desired and the actual Cartesian positions in X (right *r* = 0.9954, *p* < 0.05, left *r* = 0.9972, *p* < 0.05), Y (left *r* = 0.9995, *p* < 0.05, right *r* = 0.9986, *p* < 0.05), and Z (left *r* = 0.9979, *p* < 0.05). This shows the accuracy of the control algorithm.

**Table 2:** The table shows Mean ± Std.

**ERROR IN ANGLE**	
Right—hip flexion/extension (degrees)	1.02 ± 0.006
Left—hip flexion/extension (degrees)	0.81 ± 0.006
Right—knee flexion/extension (degrees)	0.91 ± 0.011
Left—knee flexion/extension (degrees)	1.021 ± 0.011
Left—hip abduction/adduction (degrees)	0.55 ± 0.003
**ERROR IN POSITION**	
Right—X position (cm)	0.68 ± 0.005
Left—X position (cm)	0.54 ± 0.004
Right—Y position (cm)	0.10 ± 0.0008
Left—Y position (cm)	0.13 ± 0.001
Left—Z position (cm)	0.23 ± 0.002
Time delay (s)	0.008 ± 0.0002

*Error of the (a) difference between actual and desired position (error in position) in the left and right foot in X and Y direction and in the left foot in Z direction, (b) difference between actual and desired hip and knee angle in sagittal plane for right and left leg and hip angle in coronal plane for left leg (error in angle), and (c) time delay (time for each iteration of the loop) to reach the actual position based on user input force*.

Figures 5D–F show the accuracy of the impedance control of the hip, knee (in the sagittal plane), and the hip (in the coronal plane), respectively. The actual joint angles follow the desired joint angles with minimal angular lag. This is quantitatively shown in Table 2, where the angular lag (difference between actual and desired angles) in the hip and knee (sagittal plane), and the hip (coronal plane) is <1°, and Pearson's *r* showed that correlation was high between desired and actual joint angles in hip flexion/extension (right = 0.9985, *p* < 0.05, left = 0.9984, *p* < 0.05), knee flexion/extension (left = 0.9983, *p* < 0.05, right = 0.9978, *p* < 0.05), and hip abduction/adduction (left = 0.9982, *p* < 0.05). This shows the accuracy of the impedance control.

Taken together, the results show that the biped's foot position reaches the desired position of the user with minimal lag. The time delay or control loop time (time required to move all the joints by the motor from the time the forces were applied by the user) was <10 ms (Table 2). Studies have shown that a control loop frequency of 100 Hz is sufficient for human operators to feel smooth, nearly passive, movements of a robot (Van Der Linde et al., 2002). The maximum error in the Cartesian position is <1 cm, and the error in joint angles is <1° (Table 2). These results validate that our experimental control method and robot are appropriate for the multi-participant experiments.

### Spatial and Temporal Symmetry

**Figure 6:**
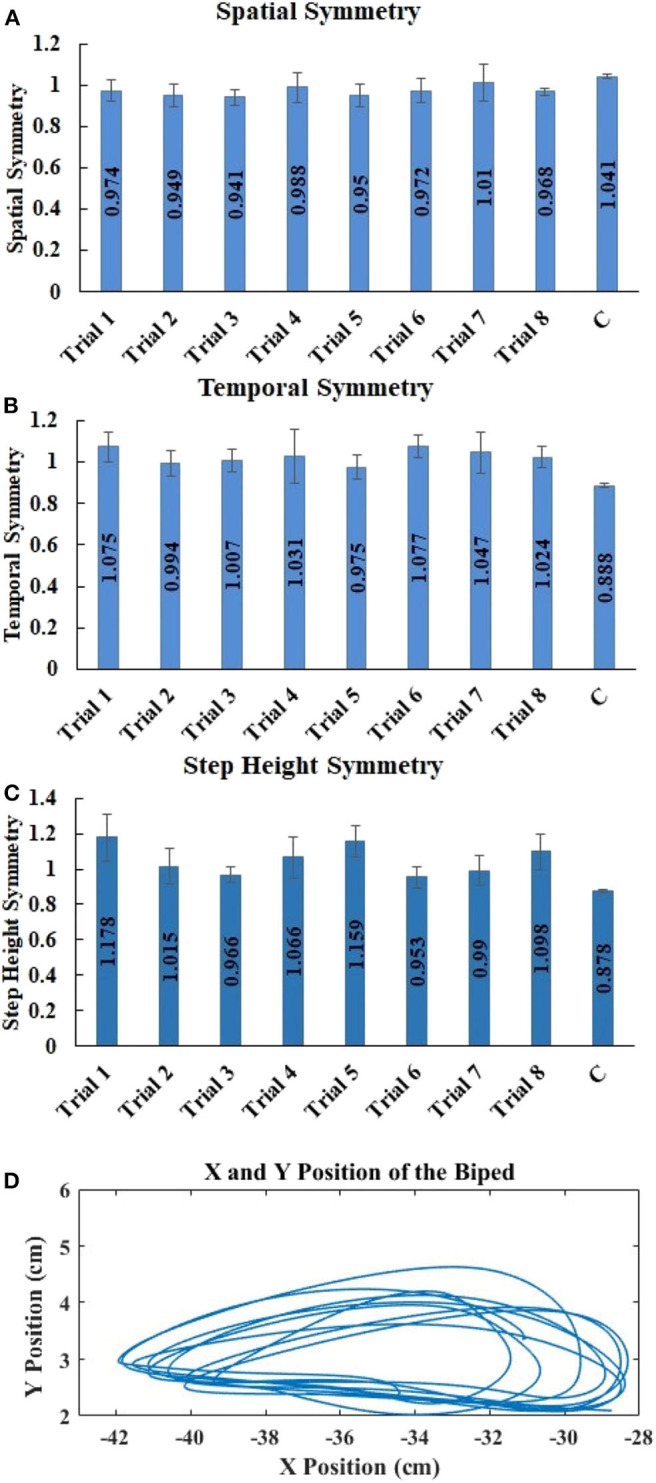
Mean ± std. error of **(A)** spatial symmetry, **(B)** temporal symmetry, and **(C)** step height symmetry, for trials 1 through 8 for biped walking by 7 participants and for 1 trial by the reference participant. **(D)** X and Y positions of one biped foot for one naïve subject walking the biped on the treadmill at medium speed.

Figures 6A–C show the overall temporal, spatial and step height symmetry, respectively, for 7 participants and 1 reference participant. All symmetries are close to 1, irrespective of speed variations, indicating that the users were controlling the biped with a bilaterally symmetrical gait. Repeated measures ANOVA shows no significant difference between trials, indicating that speed did not affect the spatial (*p* > 0.05, *F* = 0.796) and temporal (*p* > 0.05, *F* = 0.424) symmetry. In addition, Cohen's d effect size shows a low effect for both spatial (effect size = 0.21) and temporal (effect size = 0.27) symmetry, again signifying that difference between trials is very low. Friedman's test shows no significant difference between trials, indicating that speed did not affect the step height symmetry (*p* > 0.05, chi-square = 8.095). Kendall's W effect size shows a low effect for step height symmetry (effect size = 0.165), again signifying that difference between trials is very low.

### Duty Cycle

**Figure 7:**
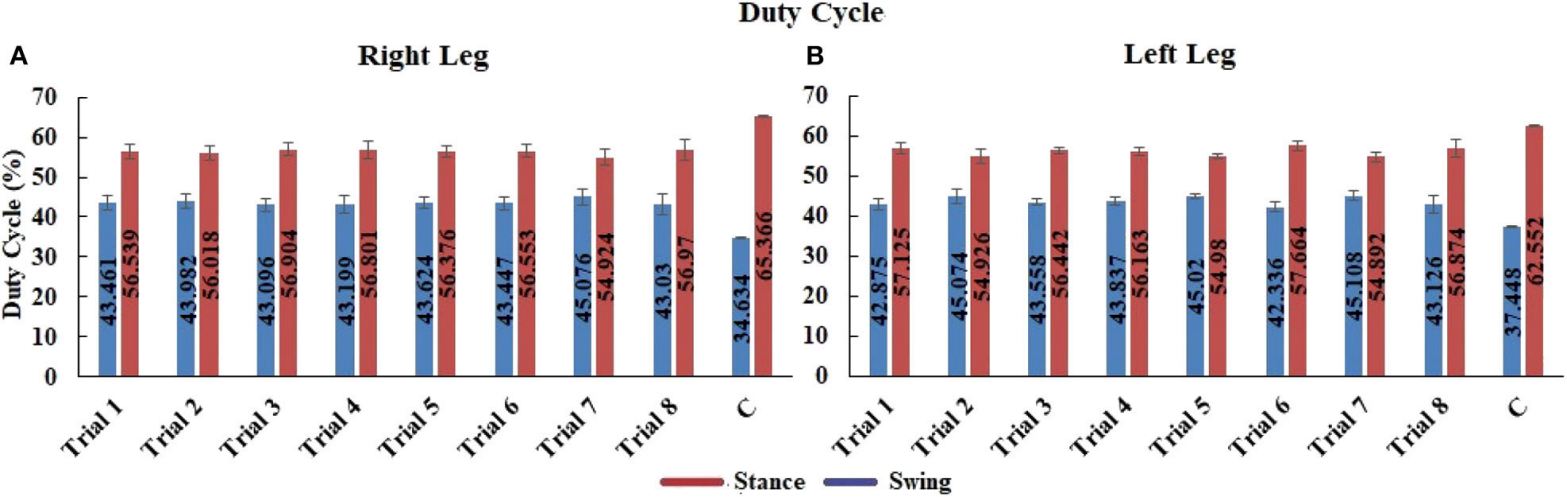
Mean ± std. error of duty cycle in **(A)** right leg and **(B)** left leg of 7 participants for trials 1 through 8, and of 1 reference participant (C) during 1 trial. Stance phase is red and swing phase is blue.

Figure 7 shows the average percentage of swing and stance phase in the gait cycle of all 7 participants for trials 1 through 8. In human gait, typical swing phase is ~40% and stance phase is 60% of the gait cycle (Winter, 2009). Our robot gait cycle across all trials was slightly over 40% swing and slightly below 60% stance. Mixed design ANOVA shows no significant difference between either trials or legs. This indicates that speed did not affect swing (*p* > 0.05, between trials *F* = 1.177) or stance (*p* > 0.05, between trials *F* = 1.177), and that swing (*p* > 0.05, between legs *F* = 0.022) and stance (*p* > 0.05, between legs *F* = 0.022) were similar in both the legs.

### Comparison of Biped and Human Gait Trajectories in the Sagittal Plane

**Figure 8:**
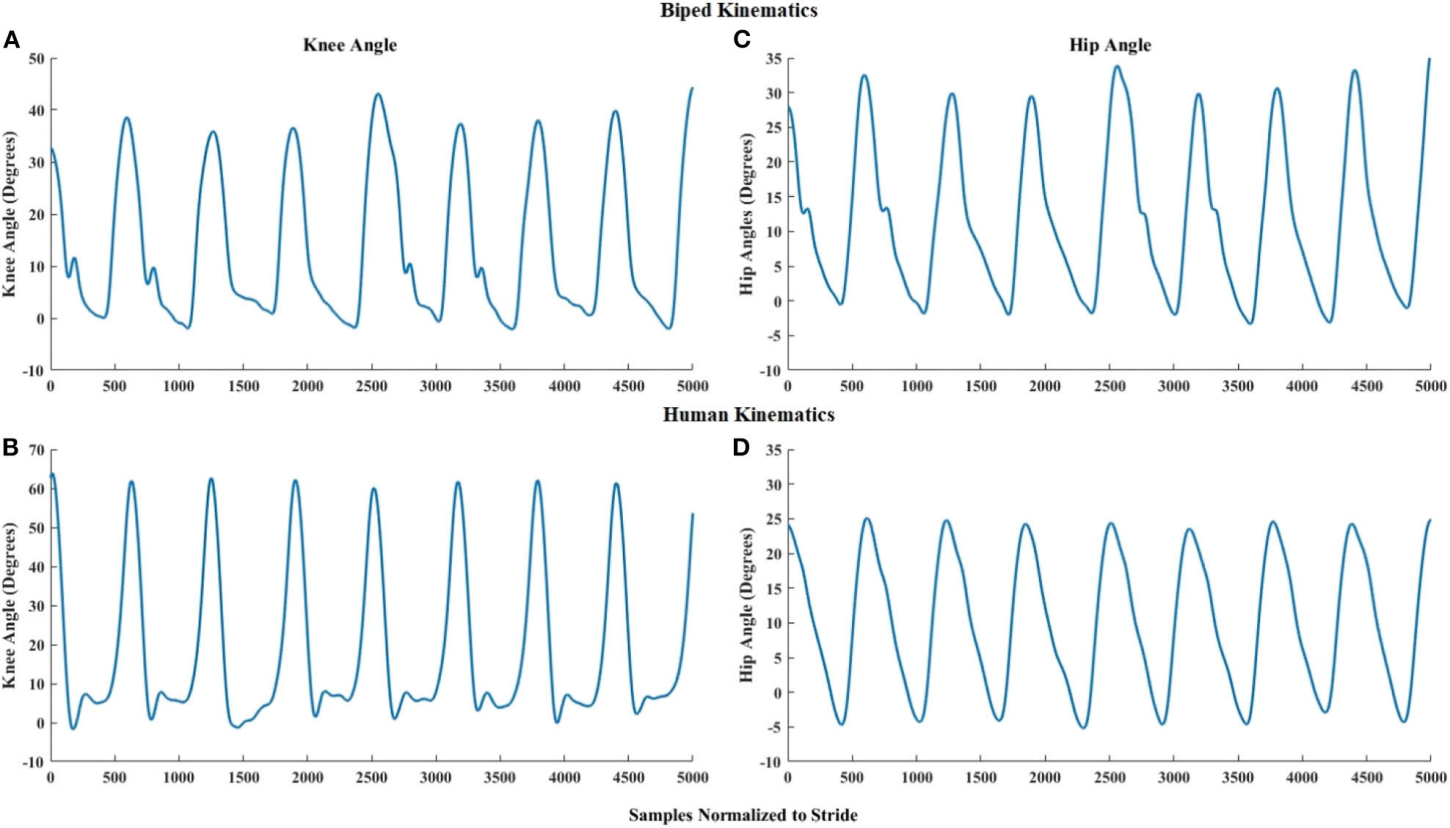
Right knee angle of multiple strides of the **(A)** user-controlled biped, and **(B)** reference participant, walking on the treadmill. Right hip angle of multiple strides of the **(C)** user-controlled biped, and **(D)** reference participant, walking on the treadmill.

Figure 8 shows a visual comparison of the hip and knee angles of a reference participant and the corresponding biped angles. The visual appearance of biped and reference participant hip and knee angles in Figure 8 is quite similar, with the human knee slightly flexing during stance. We attribute this to the knee accommodation of foot angle changes at toe-off by the human walker, while the biped maintained the foot parallel to the ground. We observe significant high positive correlations between the joint angles of the biped (for 7 participants) and the reference participant: For the knee, Pearson's mean *r* = 0.7770, std ± 0.0953, with *p* < 0.005. For the hip, Pearson's mean *r* = 0.9968, std ± 0.0926, with *p* < 0.005. This confirms that biped walking produces human-like knee and hip joint trajectories.

### User Feedback

Participant responses show that hand control was quite easy and intuitive (Table 3). The participants required little force and did not get tired from using the control method (Table 3). Six out of the seven participants responded that they felt the ground impact with their hands.

**Table 3:** The table shows the mean ± std. error for user feedback questionnaire.

**Question**	**Ease of using hand to control biped**	**Tiredness**	**Intuitiveness of hand control**	**Force required to move hands**	**Haptic feedback every time foot made contact to ground**
Options	0—easy to 10—difficult	0—not tired to 10—very tired	0—least intuitive to 10—very intuitive	0—least force to 10—large force	Yes or no
Participant responses	3.29 ± 0.52	1.14 ± 0.59	7.43 ± 1.19	1.86 ± 0.59	6 participants answered yes and 1 participant answered no

## Discussion

Lower extremity exoskeletons show potential for restoring ambulation in individuals with paraplegia due to spinal cord injury. Currently, many lower extremity exoskeletons produce a preprogrammed gait that can be initiated by the user, but not completely controlled by the user. Thus, users have limited control over their gait (i.e., step length, width, and speed), and little feedback of foot placement and ground contact. In this paper, we proposed a novel control mechanism for lower extremity exoskeletons to address these shortcomings; allowing the users to control their gait in real-time, as well as receiving haptic feedback. The accuracy and efficacy of the control mechanism was evaluated using the following outcome measures: accuracy of the foot position and joint angles; time delay between user-desired and actual robot kinematics; temporal, spatial, and step height symmetry; duty cycle of stance and swing phases; and similarity of biped hip and knee angles to those of human gait.

Our results demonstrate the effectiveness of the control mechanism, which allows the user to interactively control both legs of a ½ scale robot to produce a gait trajectory closely resembling human gait. As required for smooth real-time human-robot interaction (Van Der Linde et al., 2002), the time delay of the control mechanism is below 10 ms; ensuring smooth and stable movement. The difference between user-desired and actual robot Cartesian position and joint angles were <1 cm and 1°, respectively; showing that real-time accurate trajectories can be obtained over time by our system.

Admittance control provides the user with the following advantages: one-to-one correspondence between hand and foot movement; force amplification; intuitive control (as the user applies desired force in the intended direction of movement); and accurate time-varying trajectory control (the leg moves in the direction of the force) (Glosser and Newman, 2002; Van Der Linde et al., 2002). Research has shown that admittance control is an effective strategy for human-robot interaction, and that it requires a minimum of 100 Hz for acceptable human interaction (Glosser and Newman, 2002; Van Der Linde et al., 2002); which we have achieved.

Human gait is a rhythmic movement that is symmetrical but out-of-phase between the two legs. Hence, an effective exoskeleton control strategy should be able to reproduce the same pattern of movement over a period of time, as well as coordinate inter-limb movement (Vaughan et al., 1999; Pearson, 2003). Inter-limb coordination or symmetry is the ability to maintain temporal and spatial symmetry, which results in healthy gait (Patterson et al., 2008). Temporal and spatial symmetry close to “1,” signifies that both limbs are performing a symmetrical movement (Patterson et al., 2008). Our analysis shows that the participants controlling the biped were able to maintain inter-limb temporal, spatial, and step height symmetry close to 1 across all trials; irrespective of speed variations, and similar to that observed in human gait. Studies have shown that there is a correlation between large deviations in temporal & spatial symmetry away from 1 and falls & reduced walking speed (Balasubramanian et al., 2007; Patterson et al., 2008). Thus, we can conclude that our biped control mechanism will promote stable and safe human-exoskeleton walking. Our robot gait cycle has an average swing phase of just over 40% and a stance phase of just under 60%, as shown in Figure 7, across all trials. The deviation from the normal human gait cycle is small and likely due to the biped performing a flat-footed gait, as the foot is constrained to remain parallel to the ground, resulting in a shorter stance period. In addition, the percentage of time spent in stance and swing phase was the same between both legs, further indicating consistent temporal characteristics.

The results show that the joint angles produced by the biped (Figures 5D–F) are within the range of healthy biological gait in the sagittal plane (Winter, 2009), and that the joint angles of the reference participant and the biped are similar.

The relative horizontal and vertical excursions of biped walking were consistent with our earlier work on the benefits of haptic feedback to the hands for controlling the position of virtual feet (Karunakaran et al., 2017). More importantly, they have the same appearance as similar measures captured from unimpaired biological gait. Figure 6D shows that the right foot data from the biped (plotted as X and Y positions) bears a strong resemblance to unimpaired human foot data (Meesen et al., 2006). The user feedback questionnaire indicated that biped control was easy, intuitive, and required only minimal force.

Current commercial exoskeletons are generally slow, with small step lengths and step heights (Kozlowski et al., 2015). They usually rely on control mechanisms that initiate each step separately, which results in a delay between steps (Dellon and Matsuoka, 2007; Strickland, 2012)^1^^,^^2^ and hence a prolonged stance phase (Esquenazi et al., 2012)^1^. Our control method, on the other hand, provides continuous gait (smooth transition from swing to stance) with no delay between steps. It also provides the user with the ability to vary step length and height, based on the required speed. Thus, it produces a more natural gait in terms of temporal & spatial characteristics.

This preliminary work shows the feasibility of our algorithm to control exoskeleton kinematics and produce symmetrical gait patterns, using hands as a controller. However, there are several limitations that will need to be addressed in future work. This paper does not address balance, which will be addressed in a separate future publication. We also acknowledge that the benefits of our control mechanism must be thoroughly evaluated with participants walking independently in a full-scale exoskeleton. A wearable exoskeleton is under development for this purpose (Al Rashdan, 2016; Androwis et al., 2017).

While our work has focused on a specific SCI population of users, we are excited about expansion of the technique to individuals with other disabling conditions, such as people with diplegic cerebral palsy who have sufficient arm control to operate our trekking poles. We also see potential for the application of variations of our control mechanism in individuals with stroke and traumatic brain injury (both for assistance with ambulation, as well as for gait therapy), and multiple sclerosis.

## Conclusions

We have developed and tested an admittance-control-based user-robot control strategy that allows the user to control foot trajectories with hand-generated forces and hand-sensed foot kinematics, and thus gait in real-time. This new approach has the potential to be used with a wearable rehabilitation exoskeleton, as a control mechanism that provides users with lower leg disability complete control over gait. When implemented with wearable exoskeletons, our method has the potential to greatly improve community ambulation in individuals with lower extremity paralysis.

## Data Availability Statement

The datasets presented in this article are not available because New Jersey Institute of Technology Review Board approval does not include data sharing with external institutions.

## Ethics Statement

The studies involving human participants were reviewed and approved by New Jersey Institute of Technology. The patients/participants provided their written informed consent to participate in this study.

## Author Contributions

KK designed the algorithm, evaluated the design, analyzed the data, and drafted and finalized the manuscript. KA assisted with the design. GA reviewed the manuscript. RF assisted with the design, revised, reviewed, and finalized the manuscript. All authors contributed to the article and approved the submitted version.

## Conflict of Interest

The research for this study was conducted when the authors were employed by NJIT. Currently, KA is now employed by the company Stryker Corporation, and RF consults at Really Useful Robots, LLC, in addition to being Professor Emeritus at NJIT. The companies had no role in the design of the study; in the collection, analyses, or interpretation of data; in the writing of the manuscript; or in the decision to publish the results. The remaining authors declare that the research was conducted in the absence of any commercial or financial relationships that could be construed as a potential conflict of interest.

## Footnotes

^1^*Ekso Bionics*. Available online at: https://eksobionics.com/

^2^*RexBionics*. Available online at: https://www.rexbionics.com/

^3^*Robotis*. Available online at: http://www.robotis.com/xe/dynamixel_en

^4^https://www.universal-robots.com/
